# Microwire-Based Sensor Array for Measuring Wheel Loads of Vehicles

**DOI:** 10.3390/s19214658

**Published:** 2019-10-26

**Authors:** Jesus Olivera, Sofia Aparicio, Margarita Gonzalez Hernández, Arcady Zhukov, Rastislav Varga, Maximo Campusano, Enmanuel Echavarria, Jose Javier Anaya Velayos

**Affiliations:** 1 Laboratorio de la Dirección General de Aduanas, Carlos Sánchez, Esquina Lope de Vega, Ensanche Naco, 10119 Santo Domingo, Dominican Republic; m.campusano@dga.gov.do (M.C.); e.echavarria@dga.gov.do (E.E.); 2 Pontificia Universidad Católica Madre y Maestra, Autopista Duarte, Km 1 ½, 51000 Santiago de los Caballeros, Dominican Republic; 3 Institute for Physical and Information Technologies “Leonardo Torres Quevedo”, Department of Acoustic and Non-Destructive Evaluation, ITEFI (CSIC), 28006 Madrid, Spain; sofia.aparicio@csic.es (S.A.); m.g.hernandez@csic.es (M.G.H.); jj.anaya@csic.es (J.J.A.V.); 4 Dpto. Física de Materiales, Facultad de Química, UPV/EHU, P.O. Paseo Manuel de Lardizabal, 3, 20018 San Sebastián, Spain; arcadyzh@mail.ru; 5 IKERBASQUE, Basque Foundation for Science, 48013 Bilbao, Spain; 6 CPM–TIP, P.J. Safarik University, Tr. SNP 1, 04001 Kosice, Slovakia; rastislav.varga@upjs.sk

**Keywords:** concrete, embedded sensor, ferromagnetic microwires, switching field, SHM

## Abstract

In this paper, a magnetic microwire-based sensor array embedded under the pavement is proposed as a weighing system at customs ports of entry. This sensor is made of a cementitious material suitable for embedding within the core of concrete structures prior to curing. The objective of this research is to verify the feasibility of stress monitoring for concrete materials using an array of cement-based stress/strain sensors that have been developed using the magnetic sensing property of an embedded microwire in a cement-based composite. Test results for microwire-based sensors and gauge sensors are compared. The strain sensitivity and their linearity are investigated through experimental testing under compressive loadings. Sensors made of these materials can be designed to satisfy specific needs and reduce costs in the production of sensor aggregates with improved coupling performance, thus avoiding any disturbance to the stress state.

## 1. Introduction

Agencies for freight control, such as customs, use weigh stations to enforce weight limits, collect fees, and record the truck weight data. Within the framework of strategic objectives considered by customs, a weighing system that does not interrupt traffic is recognized, to track and supervise imports and exports products, thus ensuring that their weights correspond to the shipping company customs declaration. 

However, traditional static weigh stations are very expensive to install and operate, and require that the trucks are stopped and weighed individually. An alternative to traditional weigh stations is a weigh-in-motion (WIM) system [1,2,3,4,5] that is installed on an existing road and can estimate the weight of vehicles, avoiding possible retentions. This dynamic weighing of vehicles has several advantages, including savings in time and cost and a greater safety when operating in customs checkpoint lines. This approach can also be used for detecting overloaded vehicles, without having to go through the static weighing scale.

However, the technology based on wired sensors requires a large investment. This is due to the use of expensive commercial sensors, money and time-consuming process for embedding the sensors during the road making, and prolonged road closures during installation and maintenance. Another problem is their short lifespan. These systems can also be affected by numerous factors [6], such as load variation depending on the speed of the vehicle, roughness of the pavement, and movement of metal parts on the sensor under the pavement that can affect the sensor signal. 

The embedded sensors and systems can be essential for structural damage detection and weight control. In addition, multiple embedded sensors will be required to perform simultaneous measurements to consider the distribution of stress along the vehicle’s axles. An adequate number of sensors must be in place to form an array and measure the force applied by each tire (along its entire area) on the pavement. The deformation of the pavement under these loads causes the deformation of the sensor by means of a pressure wave propagating at sound velocity. The sensors will be calibrated on a laboratory scale and subsequently tested in real life conditions. 

Ideally, the stress measuring device length should be as short as possible to measure stress/strain in a localized zone. Concrete is considered a heterogeneous material and is only considered homogeneous on a scale of several centimeters. Therefore, the measurement of the stress in the concrete will not be easy to determine unless a good number of sensors are embedded without any disturb to the stress state of concrete itself.

For this reason, the introduction of many tiny sensors at very low cost in the interior of a concrete structure can be considered one of the most promising developments to monitor the long-term behavior of concrete structures. Moreover, they can be used to sense stress/strains and other magnitudes, such as temperature [7], humidity [8], and corrosion [9].

In this respect, amorphous glass-coated microwires are novel materials that are very promising for technological applications because of their versatile properties [10,11,12], their resistance to alkaline concentrated environments and because they may satisfy other desirable characteristics, such as small size and mass, easy coupling, high sensitivity to stress/strain, and the possibility of minimizing their sensitivity to temperature [13] and compensate other ambient conditions as spurious magnetic field [14]. Microwires are manufactured by means of a modified Taylor–Ulitovsky process [15] based on direct casting from the melt. This technique [16] allows us to control the microstructure and the diameter of nucleus and thickness of coating of the microwire resulting in different mechanical, chemical, and magnetic properties. One of the main advantages of this simple method of fabrication [17,18] is the possibility to obtain continuous pieces of microwire up to several km long from tens gram of alloy in a very short time. 

Usually, magnetic alloys require of a suitable heat treatment to acquire the desirable magnetic properties for their selected application. First all, heat treatments are necessary to stabilize the magnetic properties of the microwires over time and, moreover, heat treatments [19] are carried out for reducing the residual internal stress by means of structure relaxation. It is worth mentioning that these materials allow the structure to be more easily relaxed because of their short range order at its amorphous state [12]. The temperature of heat treatment is chosen below the crystallization temperature and is generally carried out in inert gasses such as argon or nitrogen, or in vacuum. 

The possibility of non-contact detection of the signals from the composites at high frequency is of great interest for remote non-destructive testing and structural health monitoring. Such composites consist of arrays of continuous or short-cut pieces of conductive ferromagnetic wires embedded into a dielectric matrix [20,21,22]. On the other hand, magnetic domain wall propagation at low frequency has become an important research topic because the possibility of contactless stresses measuring inside a material. A cement-based stress/strain sensor was developed by using the stress/strain sensing property of an embedded magnetic microwire in a cement-based composite (MMCC) [23]. The individual magnetic sensor consists of a magnetic microwire embedded in a cement-based material to ensure good coupling of the sensor to the concrete-based material where this sensor will be embedded. 

Recent attention has turned to the development of innovative material and composites derived from these microwires based on magnetic bistability [24,25,26,27], such as carbon or glass fiber [14], polymer composites [28] and titanium implants [29]. 

The embedded glass-coated microwires may occupy a few millimeters in length [13]. This is a contactless type sensor that measures magnetic induction variations resulting from stress variations. These sensors can be applied for weigh-in-motion measurement of individual vehicles at moderate speeds. Work in progress serves to study the impact of the depth, optimization of coils, and electronics to use this microwire system under pavements. This capability could make the weighing process more efficient because many microwires can be embedded in the pavement due to their low cost and small dimensions. In the particular case of commercial vehicles subject to customs inspection, this technology would allow for trucks under the weight limit to bypass static scales and inspection.

The objective of this research is to verify the feasibility of stress monitoring for concrete using an array of magnetic microwires and compare its performance with strain gauge sensors. Strain gauge technology is used in static weighing for direct weight enforcement and is accepted as the most accurate and reliable means to weigh a vehicle. Multiple embedded concrete strain sensors may be embedded along the road to directly measure the dynamic strain response of the pavement [30].

## 2. Experimental Section

In this work, prior to load-unload cycles in concrete specimens, microwires and MMCC sensors were characterized to assess the behavior of these components with the stress/strain measurements. 

### 2.1. Microwires

Amorphous microwires with positive magnetostriction are characterized by magnetic bistability. In the case of Fe-rich microwires with spontaneous magnetic bistability a large axially magnetized single domain is surrounded by an outer radially magnetized shell. This domain structure is determined by the internal stresses arising during the rapid solidification of the microwire. The switching between the two stable magnetic configurations (with magnetization axially aligned in positive and negative directions) appears at the switching field. The switching field depends on various external parameters, such as the magnetic field, electrical current, temperature, and mechanical stress [31,32,33]. 

The induction method is frequently used for the determination of the switching field [24]. When the excitation coil is fed by a precision triangular shape current, the switching field is proportional to the switching time in which the magnetization occurs. Once the applied magnetic field reaches the critical switching field value, the wall propagates along the microwire to its end in the direction of the applied magnetic field.

Such a method can be successfully employed in practical applications; however, the induced signal must be high enough to be distinguished from the noise. The induced signal voltage, U_i_, in one turn of the pickup coil obeys Faraday’s induction formula, which states that the electromotive force (emf) created in this coil is equal to the rate at which the magnetic flux changes through it.

U_i_ = −*∂*ϕ/*∂*t (1)

where *∂*ϕ is the variation of the magnetic flux within the time interval *∂*t. 

In the case of microwires, the variation in the magnetic flux is low due to their dimensions, but may be compensated by choosing an adequate composition of magnetic microwire that exhibits a very fast magnetization process, with *∂*t being extremely short and therefore leading to higher values of the induced signal.

A glass-coated amorphous microwire with a nominal composition of Fe_71_._7_B_13.4_Si_11_Nb_3_Ni_0.9_ glass-coated microwire with metallic nucleus diameter d = 103 μm and total diameter D = 158 μm prepared by Taylor–Ulitovsky method, was used to fabricate the MMCC sensor. The microwire length was chosen to be 6 cm, similar to the gauge length, which is important for comparing both stress/strain measurement techniques presented here. The measurement of strain is the measurement of the displacement between two points some distance apart. The gauge length can be described as the distance over which the stress/strain is averaged. The switching field and its stress dependence can be remarkably affected by all kinds of thermal treatments, for example, stress annealing [34] and conventional annealing [19], as was observed for an iron-rich amorphous microwire. The effect of annealing at 300 °C for 1 h leads to the relaxation of the strong stresses introduced during production process, as was found before in ref. [35]. This influence must be attributed to the magnetic softening previously reported, for example, for annealed FeCoMoBCu-based microwires [36]. As a result of such annealing, a lower stress is needed for the same sensor response.

### 2.2. MMCC Sensors

**Figure 1:**
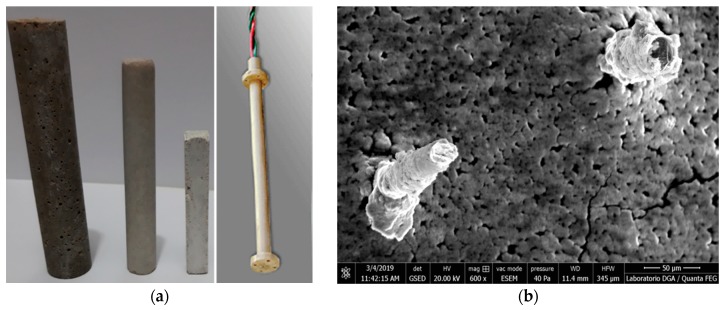
(**a**) MMCC sensors with an embedded microwire, and a strain gauge. (**b**) Electronic micrograph of the two embedded microwires.

The MMCC sensors were made of magnetic microwires embedded in mortar. Figure 1a shows three different shapes of mortar with an embedded magnetic microwire. The use of magnetic microwires allows creating a built-in stress/strain sensor inside the material without affecting its mechanical behavior. The magnetic microwire was inserted into the axis of a mortar cylinder. The cylinder geometry was chosen because the calibration of the sensor is simpler. Figure 1b shows an electronic micrograph of two embedded magnetic microwires of different diameter after polishing of the surrounding surface.

The mix proportions of the mortar are shown in Table 1. The mix proportions of the cement-based composite were selected to obtain a compressive strength similar to the aggregates used. The mortar was fabricated and stored for 24 h in a cylindrical mold with a 25 mm diameter and 64 mm length. After demolding, it was placed in immersion at 20 °C for 28 days. The process to cast and calibrate the MMCC sensor was similar to that used in [23].

### 2.3. Concrete with MMCC Sensors and Strain Gauge

**Figure 2:**
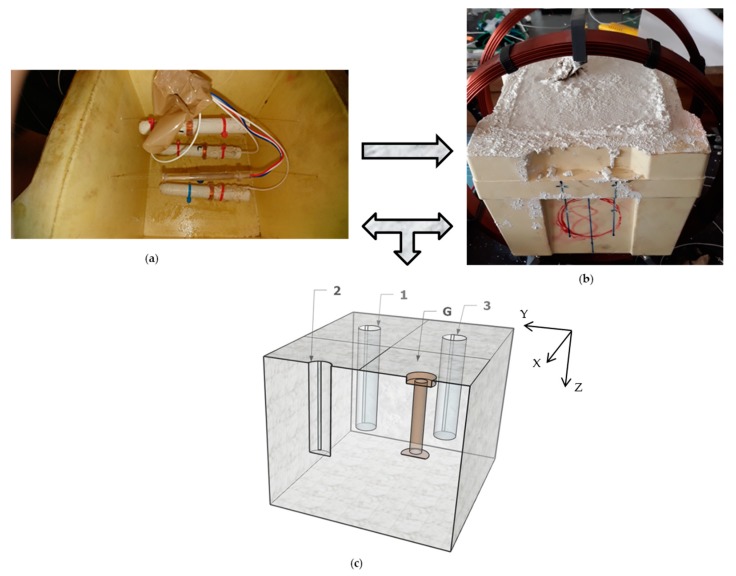
(**a**) MMCC sensors placed in the center of a normalized cubic mold of 150 mm^3^. (**b**) Concrete specimen with embedded sensors. (**c**) Schematic localization of the three sensors labeled 1, 2 and 3 and the strain gauge (G) in the concrete specimen during the stress cycles.

The MMCC sensors should be aligned parallel with the strain gauge. The three MMCC sensors, together with a strain gauge, were placed in a horizontal position parallel to the base of the square mold at the locations shown in Figure 2a. The lead wire cable of the sensor should be tied to any available support, before the concrete is poured. The sensors were fixed in their corresponding positions in the mold by means of nylon wire loops before the concrete was cast. 

The embedment strain gauge was designed for direct embedment in concrete. The gauge has a construction of the sensing element sealed into the backing made of acrylic resin for waterproofing.

The dimensions of the gauge strain sensor are 6 cm gauge length and 0.8 cm thickness, and the resistance is 120 ± 0.5 Ω. It is a uniaxial embeddable strain gauge, with an integrated temperature sensor.

The concrete was cast and stored in cubic molds of 150 mm side with the mixing proportions presented in Table 1, see Figure 2b. The concrete specimens were demolded after 24 h and cured under immersion during 28 days prior to testing. 

After demolding the specimens were rotated so that they were in the direction of the load applied with the hydraulic press during the stress cycles, Figure 2c. 

### 2.4. Test Procedures

#### 2.4.1. Instrumentation

**Figure 3:**
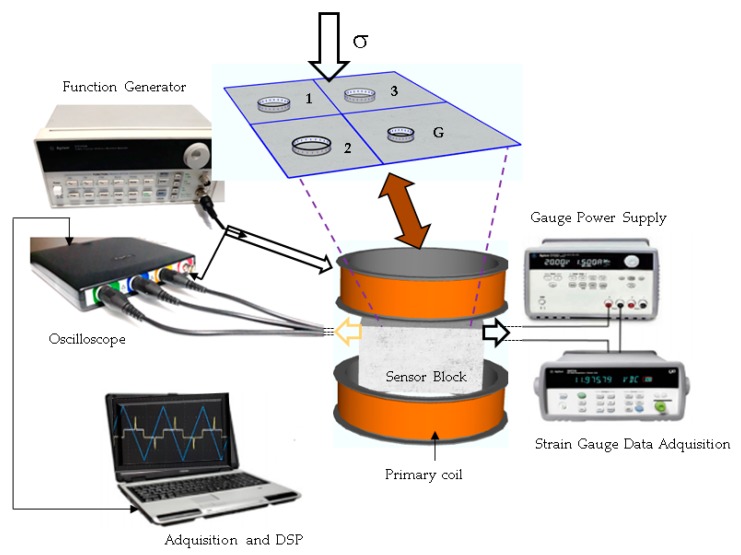
Induction method of the switching field measurement used for the experiments.

In this study, an array of three MMCC sensors and a commercial strain gauge were used for combined switching field and resistance measurements. A schematic representation of the methodology is given in Figure 3. We used the adapted Sixtus–Tonks method [37] for the calibration of the MMCC sensors. We used an excitation coil fed by a precision triangular-wave current signal to produce a linearly increasing magnetic field. In all experiments, an excitation field frequency of 600 Hz was used, and the triangular excitation field was set to 600 A/m. 

The measurements of the MMCC sensors were carried out with embedded pickup winding around the mortar cylinder and the excitation coil positioned outside. The excitation winding is connected to the power supply to generate the excitation field that magnetizes the microwire, which causes the propagation of a magnetic domain wall along the microwire. The pickup captures the induced signal where the domain wall passes through it; this signal is amplified and filtered to obtain the improved MMCC sensor signal. 

A digital oscilloscope with four channels was used to capture the signal of the sensors together with the excitation signal as a reference. 

#### 2.4.2. Stress Cycles

**Figure 4:**
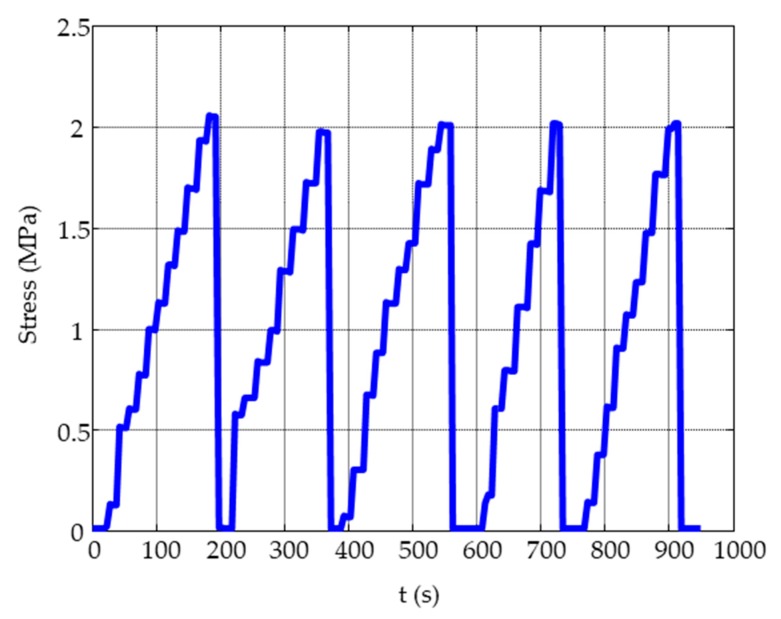
Compressive load and unload cycles applied on the concrete specimen.

The load cycles were applied with a hydraulic press. A load cell was used to measure the applied compressive mechanical force on the MMCC sensors and the strain gauge to operate below than their breaking force. Figure 4 shows the applied load and unload cycles.

#### 2.4.3. Monitoring during the Stress Cycles

**Figure 5:**
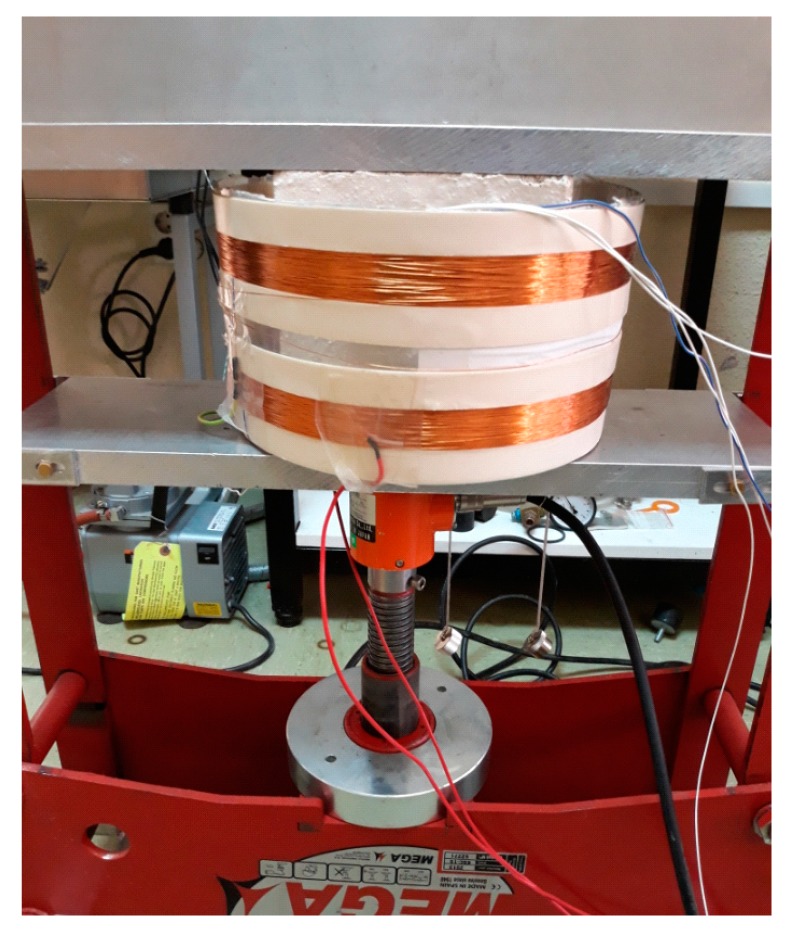
Concrete specimen with MMCC sensors and strain gauge placed in the hydraulic press.

The arrangement of MMCC sensors was described in Section 2.3, and the experimental setup was similar to that described in [23]. Figure 5 shows the setup for monitoring during the stress cycles. Based on the performance of the embedded MMCC sensor, the peak amplitude and position were measured at the value of the switching field to monitor the stress sensing properties. The strain measured by the MMCC sensor during the stress cycles was calculated based on the sharp voltage peak variations induced in the pickup coil. All measurements were recorded every 4 s along the cycles and stored in a PC for processing.

#### 2.4.4. Ultrasonic Inspection

The most commonly used non-destructive testing method to assess damage in concrete is the ultrasonic velocity measurement [38,39,40,41].

For ultrasonic inspection, a standard automatic system (with three Cartesian axes) was used. Samples were aligned at the bottom of the tank and two ultrasonic transducers (Panametrics v413, 500 kHz) in a through-transmission mode scanned the parallel surfaces of the samples with a spatial resolution of 2 mm in the horizontal and vertical direction. The setup, system and methodology used were the same as described in [39].

Two inspections were made in different faces of concrete cube, one over the plane XY, called projection azimuthal, and another on the plane XZ, called projection lateral, see Figure 2c.

Velocity and attenuation maps were generated and calculated as described in [37]. However, in this work the ultrasonic attenuation, α, is expressed in % with respect to maximum amplitude (A_m_) in the inspection. If the used gain is 40 dB, A_m_ = 1 volt; and A_s_ correspond to the maximum amplitude of the received pulse traveling through the specimen, the attenuation is:

(2) $$\alpha =\frac{A_{s}}{A_{m}}\times 100$$

## 3. Results and Discussion

### 3.1. Ultrasonic Images to Evaluate the Concrete Cube

Automated ultrasonic inspections were used to provide maps of the dimensions, velocity, and attenuation of the specimen after the compressive stress test. This inspection after the test has a two-fold purpose: on the one hand, to analyze if the structural integrity of concrete is not significantly affected by the presence of the inclusions caused by the sensors, and on the other hand, to check the final localization of the embedded MMCC sensors and strain gauge, thus providing a feedback for determining the best deposition of the sensors in the concrete specimen. 

**Figure 6:**
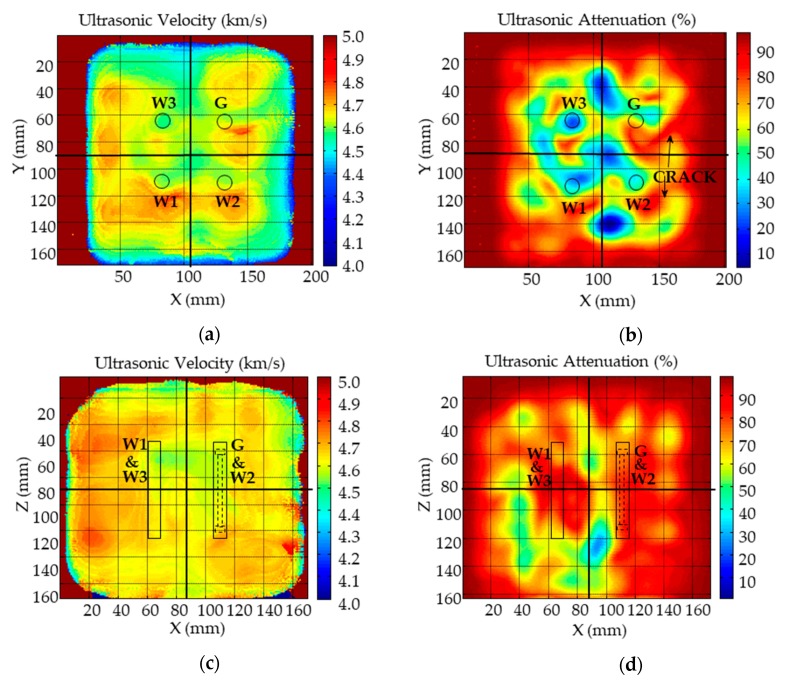
Ultrasonic images of the cubic specimen obtained after the stress cycles, (**a**) Azimuthal velocity, (**b**) Azimuthal attenuation, (**c**) Lateral velocity, (**d**) Lateral attenuation.

The images show two ultrasonic parameters, the transmission velocity and attenuation, seen from two different projections: lateral (plane XZ) and azimuthal (plane XY), as is shown in Figure 6. The color of the images is related to the value of these parameters. The velocity mainly gives information about the elastic characteristics of the material, while the attenuation clearly detects the appearance of cracks, discontinuities, or porous areas.

In the case of the azimuthal velocity image, there is a small decrease of velocity in the central zone of the specimen, where the sensors are located. However, this decrease is comparable to other areas of the specimen, and therefore the elastic properties of the material are not altered in the close vicinity of the sensor. 

However, the great attenuation that can be seen on the attenuation images, both azimuthal and lateral, may indicate that the specimen has been damaged during the tests in this area. 

It is necessary to use the two attenuation projections, lateral and azimuthal, to estimate the position and the damage extent that may have occurred during the test. To make this estimation, it is necessary to know how to interpret the ultrasonic images according to the orientation of the crack plane and the ultrasonic transmission. In general, a narrow line of high attenuation will appear when the ultrasonic transmission is parallel to the crack plane. Instead, a high attenuation zone will appear of extension similar to the surface of the cracks, when the transmission is perpendicular to the plane.

In the lateral projection image two areas of high attenuation can be distinguished, approximately one centered on the coordinates X = 60, Z = 80 and the other in the quadrant (X > 110, Z > 80). To determine if it is a crack or other type of heterogeneity, we must look at the azimuthal image. Two lines can be clearly distinguished by looking at this image, see Figure 6. Therefore, it can be considered that there are two cracks, one of which progresses from the surface to where the gauge is located. This can explain the anomalous behavior that occurred in the measurement of the gauge as will be shown below. The other crack progresses inward but does not affect the W_2_ sensor. There is another zone of high attenuation in the lateral image centered on the sensors W_1_ and W_3_, but the azimuthal image shows that this high attenuation does not appear that lines; instead, it is located in a lateral zone of the specimen, near the X = 90, Y = 160 coordinates, therefore it can be concluded that there is some heterogeneity of the surface that in principle should not affect the sensors.

### 3.2. Loading and Unloading Test over the Four Sensors

**Figure 7:**
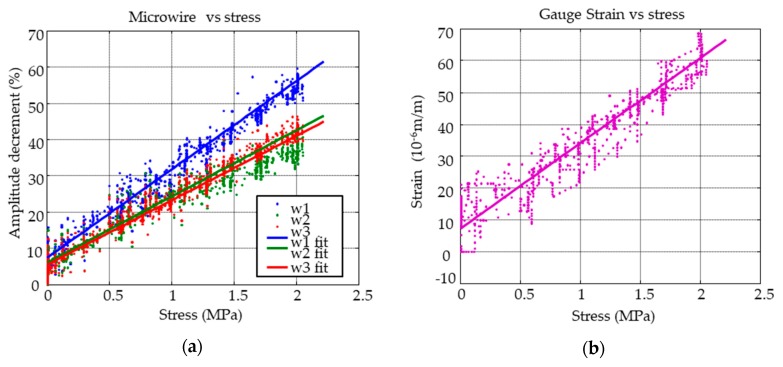
Stress measurements performed during the loading/unloading tests in the concrete specimen using (**a**) MMCC sensors and (**b**) strain gauge.

Peak amplitude decrement of the embedded MMCC sensors was the selected stress sensing property. This is shown in Figure 7a,b along with strain gauge measurements as a function of the compressive force.

At low applied stresses (<0.25 MPa), the MMCC sensors and strain gauge show almost the same linear behavior. However, the strain gauge presents more data dispersion at low compressive stresses, although the strain gauge has slightly less surface on both sides. Additionally, we suspect that at low forces, the contact pressure is not applied uniformly across the concrete cube because the force was applied with a manual hydraulic press. This condition leads to an irregular strain distribution that causes the observed deviation from the measurements of a strictly linear response. However, as soon as the metal plate is consistently pressed onto the concrete cube (usually at above 0.5 MPa compressive stresses) the measured data exhibited a proportional relationship between the contact force and sensor response. Traditionally, furnace annealing without applied stress below crystallization temperature have been employed to reduce the residual stresses, as mentioned above. The influence of this treatment on stress dependence is shown in Figure 7. One can see that thermal annealing at 300 °C increases the sensitivity about 40%. These measurements are routinely performed to calibrate each quadrant of the concrete aggregate that will be below the pavement. Therefore, each deformation value was reasonably assigned to a given stress value.

### 3.3. Results of the Strain Gauge Measurements along Load and Unload Cycles

The loading and unloading cycles were done by applying force on a single quadrant. For this we use a surface piston of 7.5 × 7.5 cm^2^. 

**Figure 8:**
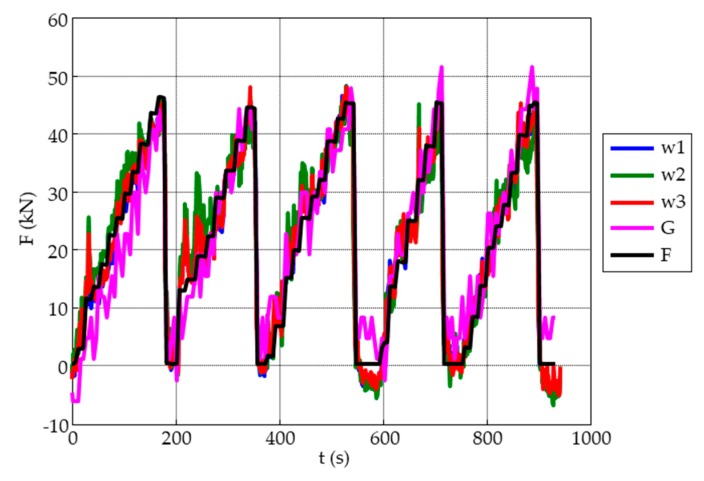
Forces measured for each embedded sensor during the loading/unloading test.

**Figure 9:**
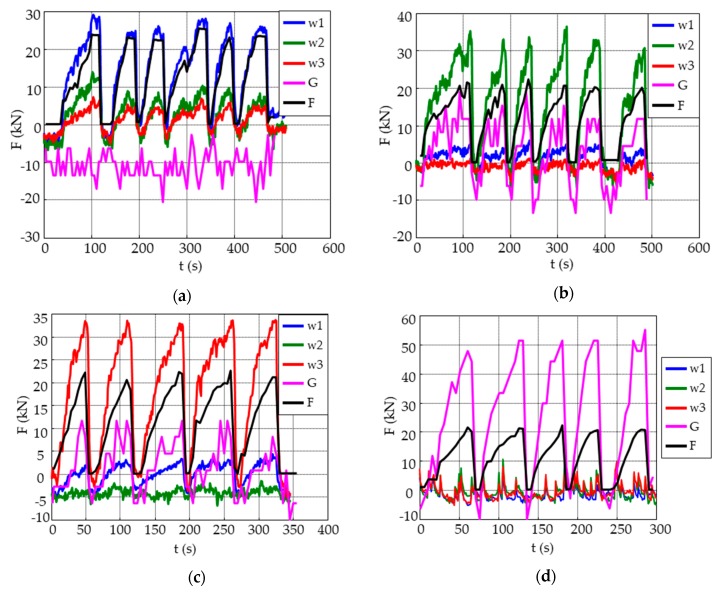
Forces measured for each embedded sensor during the loading/unloading tests on each quadrant: (**a**) W_1_, (**b**) W_2_, (**c**) W_3_, (**d**) Gauge sensors.

All plots from the loading/unloading tests (Figure 8) show a qualitatively similar response separately, as expected from the sensing principle. However, some differences can be observed. There is a response of each sensor individually and of the sensors that are in adjacent quadrants, but no signal is obtained from diagonal sensors, see Figure 9. 

However, at a quantitative level, there are large differences that should be analyzed. While the W_1_ gauge measured the applied force correctly, W_2_ and W_3_ provided overestimated values. Indeed, W_2_ and W_3_ present values of 50%, but the amount was almost 250%. It is worth mentioning that, when force was applied to the quadrant where the gauge is located, no response was obtained from the other sensors. An explanation for these results may be provided by the ultrasonic images shown in Figure 6; the area where the gauge is embedded shows a large crack that goes across almost the entire quadrant, and, to a lesser extent, the same occurs to W_2_. With respect to W_3_, no crack is observed in the ultrasonic images and the main difference is that the velocities are lower than those measured in the quadrant of W_1_.

Consequently, we provided the experimental evidence that the glass-coated microwires embedded the concrete allows monitoring of the structural health of composite material. In fact, previously a few attempts allowing monitoring of the stresses using wires (1 mm in diameter) [42] or even glass-coated microwires [27,28,29] embedded in non-magnetic matrix have been reported.

However, despite general similarity glass-coated microwire inclusions provide a few relevant features:

(i) reduced diameters (with metallic nucleus diameters from about 100 μm down to 0.05 μm);
(ii) existence of insulating glass-coating to suit particular end use requirements.

## 4. Conclusions

Measurements of stresses in concrete materials were performed using embedded magnetic microwire-based sensors and strain gauges. The entire microwire-based sensor shows a response proportional to the force; therefore, the coupling between the mortar material and the magnetic amorphous microwire can be considered good. Experimental results collected by the presented method show that it is possible to obtain a signal from the embedded microwire in the concrete composite to determine the internal stresses. However, it should be noted that once the sensors are embedded, the measured values will depend fundamentally on the characteristics of their material surrounding in every quadrant. Therefore, the system requires a calibration of each sensor separately, thus obtaining a measurement matrix ready to be embedded under the pavement. 

Hence, it is possible to estimate the structural health of composite materials and, a possible application of this microwire-based sensor array system for achieving weigh-in-motion measurements of individual vehicles at moderate speeds could be developed. These sensors can also prove their effectiveness in fatigue testing or crack detection in concrete structures at a very low cost.

In conclusion, these sensors could be manufactured at a small fraction of the cost of strain gauge sensors. Multiple sets of MMCC sensors could be embedded, being less intrusive than commercial sensors, because they can be fabricated in different shapes and sizes of mortar material. MMCC sensors provide an optimal sensitivity for the measurement of permanent and dynamical strains. 

## Figures and Tables

**Table 1:** Mix proportions of the mortar and concrete.

Materials	Mortar	Concrete
White cement I 52.5R	0.225 kg	218 kg/m^3^
Siliceous sand	0.675 kg	374 kg/m^3^
Water	0.101 kg	82.6 kg/m^3^
Gravel 0–4		255 kg/m^3^
Gravel 8–12		206 kg/m^3^
Sika Viscocrete 5990	2.2% of weight of cement	1% of weight of cement
